# Monitoring of awake bruxism by intelligent app

**DOI:** 10.12688/f1000research.110673.1

**Published:** 2022-04-29

**Authors:** Byron Velásquez Ron, Verónica Mosquera Cisneros, Pamela Pazmiño Troncoso, María Rodríguez Tates, Eddy Alvares Lalvay, Luis Chauca Bajaña, Andrea Ordoñez Balladares

**Affiliations:** 1 Prosthesis Research, Universidad de Las Américas, Quito, Quito, Pichincha, 170523, Ecuador; 2 College of Dentistry, Universidad de Guayaquil, Guayas, 090101, Ecuador

**Keywords:** Awake bruxism; self-report; ecological momentary assessment; smartphone app.

## Abstract

**Background**. Bruxism is a topic of much controversy and is continually debated in the field of dentistry due to the multifaceted clinical relationship that results in painful conditions and consequences to patients. The aim of this review was to determine the effectiveness of a smartphones app in monitoring awake bruxism.

**Methods.** PROSPERO (registration number: CRD42021271190). The eligibility criteria were as followed: observational studies, case–control   studies, studies that reported odds ratios, and studies on awake bruxism. The following keywords were searched: [smartphones apps] AND [apps] AND [awake bruxism], OR [sleep bruxism], OR [sleep hygiene], OR [parasomnias], AND [habits].

**Results**. All the authors agree that the use of the smartphone app allows controlled awake bruxism monitoring. The results also show that                                     the two bruxism are interactive, having negative synergism and substantially increasing the risks of temporomandibular joint pain and temporomandibular disorders.

**Discussion**. In the AB it was possible to identify 70% symptoms through the different frequencies of behavior provided by the App, within the present technological tools have become daily in young and adult population. The app is effective and easy to use by patients, effectively limiting biases the time of evaluation.

## Introduction

The controversy when talking about bruxism will always be latent among the academy, from a concept of parafunction to a concept of phenomena wherein biological, psychological and exogenous factors act in greater or lesser percentages. ^1^ The independent definitions of day bruxism and night bruxism were pointed out at a meeting of different specialties, with oral rehabilitation experts, maxillofacial surgeons and psychologists, who, in 2020, proposed adequate differentiation between the two. ^2^ Bruxism is a repetitive jaw muscle activity characterized by clenching or grinding of the teeth and/or by bracing or thrusting of the mandible. ^3^^,^^4^ “Bruxism has two distinct circadian manifestations: it can occur during sleep (indicated as sleep bruxism) or during wakefulness (indicated as awake bruxism). ^5^ Awake bruxism is currently defined as “masticatory muscle activity during wakefulness that is characterized by repetitive or sustained dental contact and/or reinforcements or pushes of the jaw and is not a movement disorder in healthy individuals”. ^6^

Polysomnography (PSG) and electromyography (EMG) have been used for the evaluation of nocturnal bruxism ^7^; however for the evaluation of awake bruxism (AB), there was no specific evaluator, until 2018 when an app (Manfredini, Bracci, 2018) was created to evaluate and monitor it through the use of smart devices (smartphones). ^8^ Bruxism is not necessarily considered a pathological behavior, but it has clinical consequences, the frequency of AB in the healthy young population allows us to compare with other groups ^9^; in these, psychological factors are determined, including fatigue, muscle pain, tooth wear; having differences between young people and adults differentiating habits and lifestyles that modify the behavior of bruxism. ^10^

The use of questionnaires (self-reports), such as clinical observation complemented with electromyography (EMG) have helped in the evaluation of awake bruxism; however, the momentary ecological assessment (EMA) combines real-time approaches to the current state of the patient, which facilitates having an objective assessment. ^11^ The limitations of non-instrumental methods to assess the AB are high and become subjective, the use of EMA allows to collect data in real time for a certain period of time according to the coding of alerts, which are activated according to the daily life of the individual, ^12^ the usefulness in the research field is highlighted when evaluating the oral activity of the individual, unfortunately the data obtained are partial, with little research. ^13^^,^^14^

To limit the bias provided by evaluations of the AB, a group of researchers has introduced an app (BruxApp) for smartphones, its foundation of creation is the implementation of EMA, this collects data through alerts (20 daily) with questions of related conditions simple to accept or deny by the individual: teeth in contact, habits, mandibular hypermobility, clenching and grinding of teeth; characteristic signs of AB. ^15^ It is taken as a starting point young population (young adults) ^16^ whom the researchers determine as the control group, it is monitored by the app for a week (20 daily alerts), the frequency was 28.3% in young people with AB with a low coefficient of variation in jaw muscle activity. ^17^ The objective of the present study was to determine the effectiveness of the smartphone app in monitoring awake bruxism. The PICO question was: is the application of smart apps effective in diagnosing daytime bruxism? P: Smartphone patients with the smart app. I: Intervention of all patients with bruxism C: Comparison of bruxism control with the app versus a control group. O: Observation of the percentage of bruxism control.

## Methods

**Figure 1.**
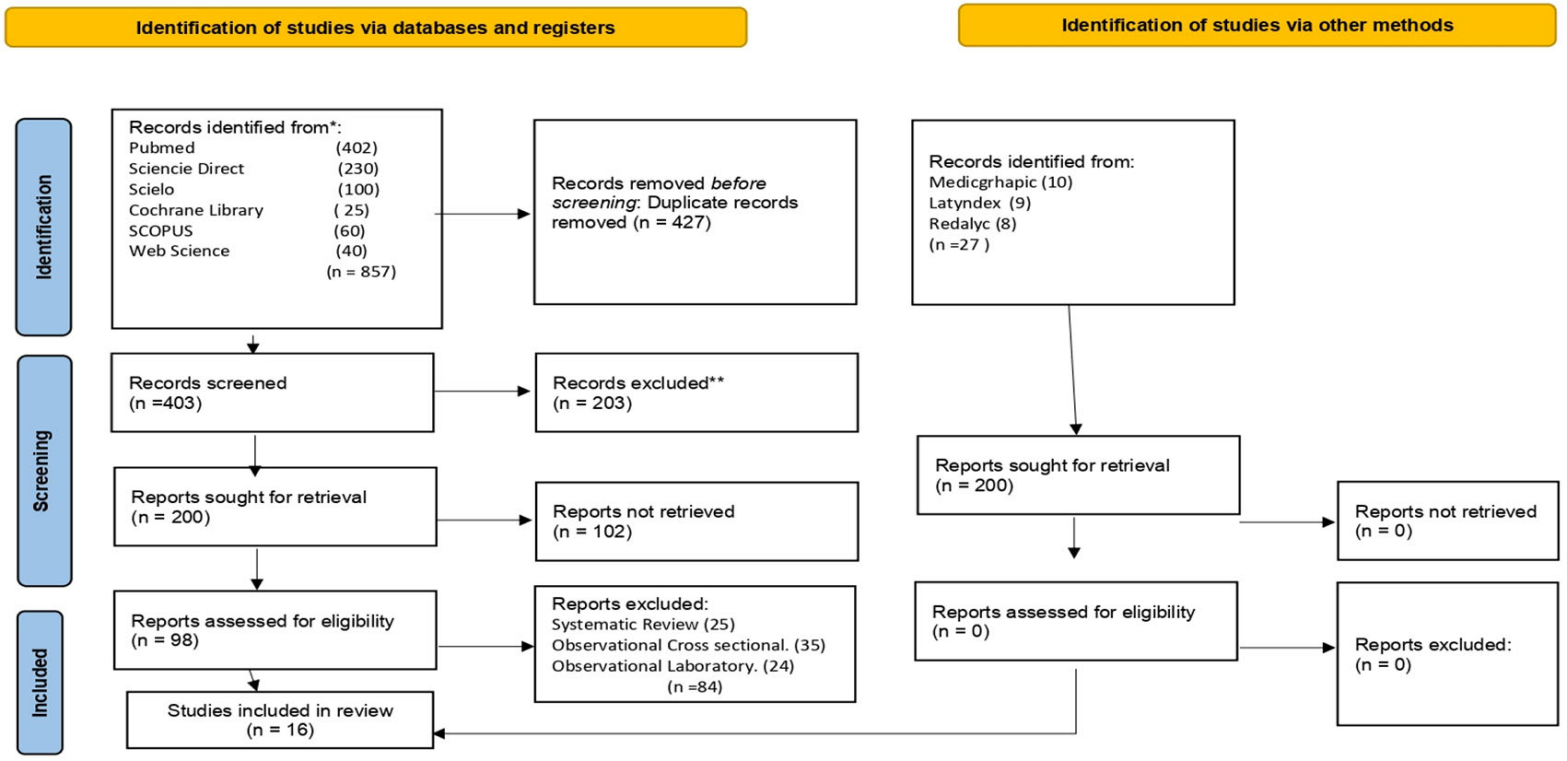
Flow diagram research.

This systematic review was registered with PROSPERO under registration number CRD42021271190. The eligibility criteria were as follows: observational studies, case-control studies, studies that reported odds ratios, and studies on awake bruxism. The following keywords were searched using the Boolean operators AND, OR and NOT: [smartphones apps] AND [apps], [awake bruxism], OR [sleep bruxism], OR [sleep hygiene], OR [parasomnias], OR [habits], OR [chewing], OR [teeth grinding], OR [squeezing teeth], OR [parafunctional habits], OR [parafunctional habit], OR [oral habits] OR [oral habit] OR [oral parafunctional] OR [oral parafunctional] OR [oral parafunctional habit] OR [oral parafunctional] OR [oral parafunctional habit] OR [oral parafunctional] OR [parafunctional oral habit] and [Facial pain] OR [temporomandibular joint disorders] OR [Temporomandibular Joint Dysfunction Syndrome] OR [myofascial pain] OR [syndromes] OR [myalgia]] OR [osteoarthritis] OR [pandemic Cov-19] OR [orofacial pain] OR [orofacial pain] OR [TMD] OR [stress] OR [temporomandibular disorder] OR [myofascial pain] OR [disk displacement] OR [young university] OR [young] OR [adult]. The Scopus, EBSCO, PubMed, Medline Embase, Cochrane Library, and Web of Science databases were searched; alternate databases that were searched included Scielo, Latindex, and Redalyc. Using the PRISMA research protocol, the authors used a flowchart to sequentially explain the selected information. The following complete articles published between January 2014 and June 2021 were included: a total of 857 records were obtained; 27 other records were obtained from other sources; 427 duplicate records were deleted; 200 studies were screened; and 102 records were excluded. In total, 98 studies were included in the qualitative analysis, and 16 studies were includedin the quantitative analysis ( Figure 1).

The authors (BVVR, VMC, PP, LCHB, EDL) independently reviewed the titles and summaries, excluded duplicates and irrelevant articles, and considered only full-text articles. The dates and names of all authors in the final review article were included. Any conflict with respect to the inclusion and exclusion criteria was resolved by the third and fourth authors (MRT, AOB). To control for bias, the Scala JADAD ( Table 1) was used. The data extraction procedure was evaluated according to the criteria of all authors. Articles were classified by the author/year, study objective, study type, methodology, results (standard mean and deviation) and conclusions.

**Table 1.** Jadad Scale for the evaluation of papers.

Study	Study described as randomized	Study Appropriate randomization and well described in the article	Study described as double-blind	Double-blind method appropriate	Double-blind description of errors	Total
Fluerasu, M. et al. 2020. ^13^	yes	yes	yes	yes	yes	5
Mir Faeq Ali Quadri, et al. 2015. ^22^	yes	yes	yes	yes	yes	5
Bracci, et al. 2018. ^23^	yes	yes	yes	yes	yes	5
Reissmann, D. et al. 2017. ^24^	yes	yes	yes	yes	no	4
Machado, N. et al. 2020. ^25^	yes	yes	yes	yes	yes	5
Zani, A. et al. 2019. ^30^	yes	yes	yes	yes	yes	5
Imbriglio, V. et al. 2020. ^32^	yes	yes	yes	yes	yes	5
Sierwald, I. et al. 2015. ^34^	yes	yes	no	yes	yes	4
Wetsellaar, P. et al. 2021. ^41^	yes	yes	yes	yes	yes	5
Wetselaar, P. et al. 2019. ^42^	yes	yes	yes	yes	yes	5
Somay, E. et al. 2021. ^43^	yes	yes	no	yes	yes	4
Zani, A. et al. 2021. ^44^	yes	yes	yes	yes	yes	5
Rofaeel, M. et al. 2020. ^46^	yes	no	yes	yes	yes	4
Serra-Negra, J. et al. 2021. ^47^	yes	yes	yes	yes	yes	5
Winocur, E. et al. 2019. ^48^	yes	no	yes	yes	yes	4
Osiewicz, M. et al. 2018. ^50^	yes	yes	yes	yes	yes	5

## Results

SB is related to nonfunctional occlusion, while AB is related to occlusal interactions, suggesting the need for a different therapeutic approach ( Table 2).

**Table 2.** Summary review.

Article/Author	Country	Objective/Study Type	Participants	Folow up of period	Results	Conclusion
Association of Awake Bruxism with Khat, Coffee, Tobacco, and Stress among Jazan University Students Mir Faeq Ali Quadri, et al. ^22^ 2015	Saudi Arabia	To assess the prevalence of bruxism among university students cross-sectional descriptive	response percentage (95%), questionnaire in one hour. 85% (63%, men; 22%, women)	14 days	Regression analysis revealed an association of stress (P = 0.00; OR = 5.902, 95% CI 2.614–13.325) and khat use (P = 0.05; OR = 1.629, 95% CI 0.360–7.368) with bruxism. Interestingly, it is noted that the khat chewer experienced 3.56 times (95% CI; 2.62-11.22) less pain compared to non-users.	When evaluating the association of bruxism with chewing using Khat (Catha edulis) important risk indicators were observed due to their association.
Association of temporomandibular disorder pain with awake and sleep bruxism in adults Wetselaar, P. et al. ^43^ 2019	Netherlands	To assess the association of TMD pain with awake and sleep bruxism in adults. Case control	733 patients with TMD (mean age 41.4; 16.3 years); 82% women diagnosed with TMD (RDC/TMD) 890 subjects (mean age 40.4; 11.8 years); were evaluated 57% women) without TMD	2 months	11.2% of controls reported squeezing or grinding while awake, this proportion was significantly higher in patients with TMD (33.9%; p<0.001). Nocturnal squeezing 23.5% of controls and 49.4% of patients with TMD (p<0.001). TMD pain risk was not significant for AB (OR 1.7; CI 1.0–2.7) sleep bruxism (OR 1.8; CI 1.4–2.4). The risk of TMD pain increased substantially in cases of simultaneous presence of awake and sleepy bruxism (OR 7.7; CI 5.4–11.1).	Awake bruxism and sleep bruxism significantly increases the risk of pain in TMD. Apparently, the two bruxism are interactive, they have negative synergism that substantially increases the risk of TMD pain.
Frequency of awake bruxism behavior’s in the natural environment. A 7-day, multiple-point Observation of real-time report in healthy young adults. Bracci, A. et al. ^19^ 2020	Italy	Assess awake bruxism (AB) behaviors in a sample of healthy young adults using a smartphone-based app for real-time reporting (i.e., momentary ecological assessment [EMA], also called experience sampling method [ESM]) Case Control	46 dental students used a phone app on smartphones sends 15 alerts at random intervals during the day in 1 week to collect self-reports AB.	7 days	Relaxed jaw muscles, during the 7 days, was 71.7%. Tooth contact (14.5%) and jaw clenching (10.0%) were the most frequent AB behaviors. No significant gender differences were detected.	The average frequency of the different behaviors of awake bruxism (contact between teeth; clenching of teeth; grinding of teeth; clenching of the jaw), measured as a function of the percentage of "positive alerts" during a 1-week observation period in the study sample, is 28.3%.
Interaction Between Awake and Sleep Bruxism Is Associated with Increased Presence of Painful Temporomandibular Reissmann, D. et al. ^46^ 2017	Germany	To explore whether AB and SB interact in their associations with painful temporomandibular disorders (TMD) and whether the interaction is multiplicative or additive Case Control	Multicenter Validation Project with a sample of n = 705	7 days	Based on age- and sex-adjusted logistic regression analyses, the main effects for both awake bruxism (OR = 6.7; 95% CI 3.4 to 12.9) and sleep (OR = 5.1; 95% CI 3.1 to 8.3). While the multiplicative interaction (OR = 0.57; 95% CI 0.24 to 1.4) was not significant, the results indicated a significant positive additive interaction (RERI = 8.6; 95% CI 1.0 to 19.7) on the OR scale.	Awake and sleep bruxism are associated with a greater presence of painful TMDs, both types of bruxism are not associated independently, but interact additively. As such, the presence of each factor amplifies the effect of the other.
Temporomandibular joint and oclusal changes in subjects with awake and sleep bruxism Fluerasu, M. et al. ^41^ 2020	Rumania	To determine an association between bruxism (sleeping and awake), occlusion (static and dynamic) and pain medications in TMJ in healthy adults Cross- sectional study	60 subjects (33 women and 27 men), 30 of them with bruxism, were investigated. Bruxism with joint pain, muscle and/or fatigue, absence of central relationship, dental wear, in static occlusion. Dynamic occlusion was analyzed as well.	1 month	Muscle pain and the feeling of fatigue of the chewing apparatus were higher in subjects with bruxism than in those without bruxism (3.23 vs 1.46). Joint noises and joint pain occurred more frequently in subjects with bruxism. Sleep bruxism is associated with lateral change of the jaw during mouth opening, joint pain, and non-functional lateral orientation. A lack of occlusal involvement was found in the etiology of awake bruxism.	Sleep bruxism is related to non-functional occlusion, while awake bruxism showed occlusal interaction, suggesting the need for a different therapeutic approach.
The association of self-reported awake bruxism with anxiety, depression, pain threshold at pressure, pain vigilance, and quality of life in patients undergoing orthodontic treatment Machado, N. et al. ^25^ 2020	Brazil	To assess whether the presence of awake bruxism was associated with TMD symptoms, pressure pain threshold, pain monitoring, oral health-related quality of life (OHRQoL), anxiety symptoms and depression in people undergoing orthodontic treatment. Cross- sectional study	Sample divided into two main groups according to the presence (n=56) and absence (n=58) of possible awake bruxism. Multi-way analysis of variance (ANOVA) was applied (p=0.050).	6 months	No TMJ and/or muscle pain were observed in both groups. Time, sex, age group, and awake bruxism did not affect PPT in masticatory muscles and pain surveillance (p>0.050). However, the primary effect of awake bruxism was observed when anxiety (ANOVA: Levels of F=8.61, p=0.004) and depression (ANOVA: F=6.48, p=0.012) were higher and OHRQoL was lower (ANOVA: F=8.61, p=0.004).	Patients with awake bruxism undergoing orthodontic treatment did not develop masticatory muscle pain/TMN. Awake bruxism is associated with high levels of anxiety, depression, and poorer OHRQoL during orthodontic treatment.
The prevalence of awake bruxism and sleep bruxism in the Dutch adolescent population Wetsellaar, P. et al. ^42^2021	Netherlands	To assess the prevalence of awake bruxism and sleep bruxism in the Dutch adolescent population. Case control	Sample of 920 subjects questioned about their bruxism behavior during the day and during sleep. Two age groups (for 17 and 23 years, respectively), gender and socioeconomic status.	12 months	A prevalence of 4.1% and 4.2% was found for awake bruxism and 7.6% and 13.2% for sleep bruxism. Women reported awake bruxism more frequently than men in the 17-year-old age group (5.0% and 3.2%, respectively), while in the 23-year-old age group it was the other way around (4.0% and 4.4%, respectively). Regarding bruxism sleep, women reported higher percentages than men in both age groups (7.8% versus 7.5% and 14.9% vs. 11.5%, respectively). On socioeconomic status (SES), awake bruxism was found more frequently in the high SES groups (4.6% versus 3.7% and 4.9% versus 4.0% in both age groups, respectively), as well as for sleep bruxism in the 23-year-old group (16.5% vs. 8.6%). In the 17-year-old group, sleep bruxism was most frequently reported in the low SES group (9.7% vs. 5.3%).	Sleep bruxism is a common condition in the Dutch adolescent population, while awake bruxism is rarer.
Evaluation of Sleep Bruxism and Temporomandibular Disorders in Patients Undergoing Hemodialysis Somay, E. et al. ^43^ 2021	Turkey	It assessed the incidence of sleep bruxism and TMD in patients undergoing hemodialysis and compared it with that of healthy individuals. Case control	Sample of 137 patients of which 68 on hemodialysis and 69 healthy individuals. They filled out TMD questionnaire. Muscle examination The level of significance was determined in p<0.05.	6 months	A high response rate (95%) was obtained since the distribution and collection of the questionnaire was within an interval of one hour. 85% (63%, men; 22%, women) experienced an episode of bruxism at least once in the past six months. Regression analysis revealed an association of stress (P = 0.00; OR = 5.902, 95% CI 2.614–13.325) and khat use (P = 0.05; OR = 1.629, 95% CI 0.360–7.368) with bruxism. Interestingly, it is noted that the khat chewer experienced 3.56 times (95% CI; 2.62-11.22) less pain compared to non-users.	It can be concluded that hemodialysis patients are more sensitive to TMDs, sleep bruxism, and related dental health problems than healthy individuals.
Music Modulates Awake Bruxism in Chronic Painful Temporomandibular Disorders Imbriglio, V.et al. ^32^ 2020	Canada	Determine if listening to a musical intervention (relaxing music), modulates the activity of the masticatory muscles and for this the electromyography activity (EMG) Case Control was recorded	14 women with chronic mTMD and 15 women without any involvement; plus a control group without musical intervention duration of 15 minutes. It was analyzed with each of the groups contrasting the difference between groups.	1 month	In both groups, the EMG position was significantly affected by the experimental block interaction group (p <0.001) compared to pink noise (mean (95% CI). EMG position increases during stressful music blocking. The EMG position was higher in individuals with mTMD than in controls during favorite music. In mTMD participants, compared to inappropriate music generating EMG block, bruxism increased during stressful music blocking.	Relaxing music was shown to model the activity of the masseter muscles, in contrast to noisy music that increases the activity of the masseter muscle (awake bruxism).
The intensity of awake bruxism episodes is increased in individuals with high trait anxiety Rofaeel, M. et al. ^46^ 2020	Canada	Measure massage activity and duration intensity of spontaneous episodes of gritting in healthy individuals with different levels of trait anxiety (TA). Case Control	A sample of 2993 Israeli high school students, from five high schools, completed online questionnaires on sleep-wake bruxism, emotional aspects, smoking, alcohol consumption, oral habits, and facial pain, and masticatory alterations. The final sample of the study on awake and sleeping bruxism included 2,347 participants.	1 month	Masseter activity was higher in the high BP groups (10.23 ± 0.16% MVC) than in the intermediate TA groups (8.49 ± 0.16% MVC) and low (7.97 ± 0.22% MVC) (all p <0,001). All groups p="">0.05). The EMG amplitude of tooth clenching episodes was higher in the high BP groups (19.97 ± 0.21% CVS) than in the intermediate <0, 05).></0, 05).> (16.40 ± 0.24% CVS) and low (15.48 ± 0.38% MVC) BP groups (all p</0,001).> The cumulative duration of fist-clenching episodes was not different between groups (p=0.390).	Among adolescents, sleep and wakefulness bruxism are associated with both emotional aspects and symptoms of facial pain and/or alterations of the masticatory system.
Smartphone based assessment of awake bruxism behaviors in a sample of healthy young adults finding from two university’s center Zani, A. et al. ^45^ 2021	Italy	Assess the frequency of AB behaviors by adopting EMA smartphone-based technology for one week in a sample of healthy young adults recruited from two different colleges. Case Control	In a sample of 255 people who completed a web survey. Using their anxiety disorder (AT) scores.	6 months	The prevalence of bruxism in the two groups (normal and HFS) was not significantly different (p=0.37). The rate was not significantly different between sleeping and awake bruxism (p=0.15) in both groups. Stress influenced the occurrence of bruxism in these two groups (p<0.001).	The intensity of episodes of awake bruxism increases in individuals with a high trait of anxiety.
Self-reported awake bruxism and chronotype profile a multicenter study in Brazilian, Portuguese and Italian dental students. Serra-Negra, J. et al. ^48^ 2021	Brazil	To assess the association between self-reported awake bruxism (AB) and chronotype profile among Brazilian, Portuguese and Italian dental students Case control	Patients with hemi facial spasms (HFS) were enrolled in the department of clinical neurophysiology for a period of 6 months.	1 week	The prevalence of awake bruxism in all groups was 33.7%. The intermediate chronotype profile was the most prevalent (60.4%), and only 16.7% of the participants had the morning profile. Univariate analysis showed that older dental students (OR = 2,640, 95% CI 1,388–5,021) and those with the uniformity chronotype profile (OR = 3,370, 95% CI 1,302–8,725) are associated with awake bruxism.	The results of this study showed that, although stress has been described as one of the most common aggravating factors in patients with bruxism, and that even if stress is predominant in the HFS group, patients with hemi facial spasm do not present bruxism more than the general population.
The prevalence of wake bruxism and sleep bruxism in the Dutch adult population Wetsellaar, P. et al. ^42^ 2021	Netherlands	To assess the prevalence of awake bruxism and sleep bruxism in the Dutch adult population. Case Control	One hundred and fifty-three (N = 153) healthy young adults (mean +/- age SD = 22.9 +/- 3.2 years).	7 days	A prevalence of 5.0% of the total population was found for awake bruxism and 16.5% for sleep bruxism. As for the five age groups, a prevalence of 6.5%, 7.8%, 4.0%, 3.2% and 3.0%, respectively, was found for awake bruxism, EPISODE computer sofwear	Information on the frequency of different awake bruxism behaviors was provided by adopting the EMA approach. Thanks to the use of Smartphone technology. about 23.6% presented awake bruxism behavior and the most frequent condition was "contact with the teeth", with a percentage of 13.6%
Awake and sleeping bruxism among Israeli adolescents. Winocur, E. et al. ^49^ 2019	Israel	To determine the emotional, behavioral, and physiological associations of sleep and awakened bruxism among Israeli adolescents. Case Control	Sample of 255 people who completed a web survey.	1 year	(43.4%) participants reported experiencing no form of bruxism (neither sleep nor awake), (34.5%) reported awake bruxism, (14.8%) reported sleep bruxism, and (7.3%) reported both sleep and awake bruxism. The onset of sleep bruxism was anxiety (mild, moderate and severe anxiety, Odds Ratios (OR) of 1.38, 2.08 and 2.35, respectively). Stress increased the risk of SB by 3.2%, temporomandibular symptoms (OR = 2.17) and chewing difficulties (OR = 2.35). Neck pain showed a negative association (OR = 0.086). Multivariate analyses for awake bruxism showed an effect of moderate anxiety (OR = 1.6).	Anxiety is considered an important trait in patients suffering from awake bruxism. Electromyography is used to measure episodes of spontaneous tooth tightening during wakefulness, it was shown that healthy individuals with a high and clinically relevant anxiety trait have increased mass activity and more intense spontaneous episodes of teeth clenching upon awakening.
Ecological Momentary Assessment and Intervention Principles for the Study of Awake Bruxism Behaviors, Part 1: General Principles and Preliminary Data on Healthy Young Italian Adults Zani, A. et al. ^30^ 2019	Italy	Discuss the general principles of EMA and EMI (Momentary Ecological Intervention) and comment on a preliminary dataset collected with smartphone app in Italian youth population. Case Control	A cross-sectional study involving 205 dental students was conducted.	7 days	During the first 7 days (T1), the average frequency of reports of relaxed jaw muscles at the population level was 62%. Contact with teeth (20%) and braces (14%) were the most frequent AB behaviors. No significant gender differences were detected. A month later, during the second week of data collection (T2), the frequency of conditions was as follows: relaxed jaw muscles 74%, teeth in contact with 11% and jaw bracing 13%.	The chronotype profile is based on inter individual differences with respect to the wake-sleep rhythm and the preferred time to perform various activities. Students over the age of 22 and those with the evening chronotype profile were the most likely to suffer from sleep bruxism.
Bruxism Behaviors Part 2: Development of a smartphone application for a multicenter investigation and chronological translation for the polish version Bracci, A. et al. ^23^ 2018	Italy	Describe the process of understanding the BruxApp smartphone application in the context of an ongoing multicenter project on the epidemiology of awakened bruxism (AB). Case Control	Sample of healthy young adults, dental students from 11 universities.	7 days	There are two software versions available, namely BruxApp and BruxApp Research. For both versions, a reverse translation was performed from Polish to English to verify the accuracy of the translation procedure. The validity of the translation has been confirmed by the perfect agreement between the original and back-translated versions in English, and the Polish version of BruxApp can thus be introduced into the clinical and research environment to deepen the study of AB epidemiology in Poland.	Sleep bruxism is a common condition in the Dutch adolescent population, while waking bruxism is rarer.

## Discussion

All the authors agree that the use of the smartphone app allows controlled AB monitoring by the patient. The current study also showed that the two bruxism are interactive, with negative synergism substantially increasing the risks of TMJ pain and TMD. Signs such as contact between the teeth, clenching of teeth, teeth grinding, and jaw clenching are well defined in the applicationIn AB, it was possible to identify 70% symptoms through the different frequencies of behavior provided by the app, within the present technological tools have become daily in young and adult population. ^18^ In the studies reviewed, the EMA was clear for the entire assigned sample. ^19^^,^^20^ In the studies that entered the analysis, the six conditions indicated by the application menu were investigated, relaxed jaw muscles (non-contact teeth), teeth in contact (sander in fixed position), mandibular clenching (without contact between the teeth), dental clenching (strong contact in fixed position), dental grinding and area of pain (temporary, interciliary, temple, preauricular, auricular, mandibular angle, mentonian, neck, frontal, infra and supraorbital), ^21^^,^^22^ the data that were obtained were handled by the application menu that allowed to precisely extract a Microsoft Excel file (20 alerts × 7 days) in real time. ^23^ The limitation that was found in the present systematic review is the difficulty of comparing with other studies by the different experimental designs (retrospective), while to apply self-reports are unique times. ^24^ By assessing population behavior frequency is the baseline for observational EMA studies that aids massive data collection, ^25^ it also helps to compare findings related to dietary habits, smoking, medications, psychological pathologies, and comorbid conditions. ^26^ Some studies take as a control group young population Bracci *et al*. 2018 analyzed healthy young population finding dental contact (13.6%), teeth grinding (0.5%) and relaxed jaw muscles (76.4%), with a combined frequency of AB of (23.6%). ^26^ Some studies take as a control group young population Bracci *et al*. 2018 analyzed healthy young population finding dental contact (13.6%), teeth grinding (0.5%) and relaxed jaw muscles (76.4%), with a combined frequency of AB of 23.6%. ^26^ These results could be considered a reference point for future research on the epidemiological characteristics of AB in healthy young adults, young people with pathologies, adults and geriatric patients. ^16^^,^^27^ The importance of psychological factors was determined, well-defined changes after the COVID-19 pandemic, having been analyzed in AB, the findings were that females are more likely to experience stress, compared with males, the explanation women report better about their emotions ^28^ but the depressive state leads to generate AB crisis with BS in the two genders due to the socio-economic conditions generated by the pandemic, it should be clarified that previous systematic reviews found no gender differences in the frequency of AB ^29^ which contrasts with current information. ^30^ No significant differences were found in the university population, young adults, some authors point out that the monitoring could have been carried out in transition for the student population so that high stress was not indicated, it would be important to develop future research in times such as semester evaluations to determine significant differences. ^31^ It should be considered that the elaboration of the self-report must be controlled, so that unnecessary biases are avoided, for this reason the calibration of the instrument is essential whether individual or group, avoiding or reducing homogeneity to a minimum, ^32^^,^^33^ through training and socialization that allows the population to understand the reliable use of self-report based on EMA. ^34^ The characteristics of the populations studied directly influence the results, the age factor, educational level, work activity, socioeconomic status are aspects that influence in substance. ^34^^,^^35^ Zanni *et al*. 2019 found that in one week the relaxation of the mandibular muscles was very low, they conclude that not only in healthy young population the symptoms change from one day to the next, ^36^ the population comportment must be specific, this makes variable the behavior of the AB monitored with the app, recognizing natural fluctuation and difficulty in recognizing the symptoms. ^37^^,^^38^ Muscle relaxation can be recognized by the individual, also clenching of teeth, ^39^^,^^40^ can be a good reference to evaluate the behavior of AB to be a conscious and controlled activity, ^41^ other authors indicate that the use of SMEs provides reliability in the monitoring of AB, the reason lowers the influence of natural fluctuation that the population presents regardless of age or gender. ^42^ It is recommended to conduct future research that considers long-term monitoring of AB, the hypothesis should be tested that the manifestations of AB: relaxed jaw muscles (non-contact teeth), teeth in contact (sander in fixed position), mandibular clenching (no contact between teeth), dental clenching (strong contact in fixed position), dental grinding and area of pain (temporary, interciliary, temple, preauricular, auricular, mandibular angle, mentonian, neck, frontal, infra and supraorbital, clinical consequences such as temporo mandibular joint dysfunction, regional myalgias ^43^^,^^44^ are determined. Continuing with the technological line, the effectiveness of an email-based registration and recovery system should be studied if the individual detects non-functional diurnal contact or muscle contracture, an effective strategy for the treatment of temporo mandibular disorders. ^45^ An assessment of the associated factors and conditions can, in theory, increase or decrease the frequencies of AB behaviors in the app monitored population based on the EMA self-report ( *e.g.*, dietary, or smoking habits, medication use, psychological problems, and comorbid conditions). ^47^^,^^48^ Data can be added to ongoing studies that consider the 2018 definition of bruxism ^49^ and the refinement of assessment strategies. Comparisons between populations are necessary and can be used in the context of an ongoing multicenter project on the epidemiology of bruxism. ^50^

## Conclusions

The app used to monitor awake bruxism is effective, and its ease of use allows a fundamental approach to diagnosis.

## Author contributions

**Velasquez B:** Conceptualization, Data Curation, Formal Analysis, Investigation, Methodology, Project Administration, Resources, Validation, Visualization, Writing Original Draft Preparation. Writing -review & Edith.

**Alvarez E.:** Conceptualization, Data Curation, Formal Analysis, Investigation, Methodology, Project Administration, Resources

**Mosquera V:** conceptualization, Data Curation, Formal Analysis, Investigation, Methodology, Project Administration, Resources

**Pazmiño P:** conceptualization, Data Curation, Formal Analysis, Investigation, Methodology, Project Administration, Resources

**Rodriguez M:** Conceptualization, Data Curation, Validation, Visualization, Writing – Original Draft Preparation, Writing – Review & Editing;

**Chauca L:** Formal Analysis, Resources, Supervision, Validation, Visualization, Writing – Original Draft Preparation, Writing – Review & Editing

**Ordoñez A:** Formal Analysis, Resources, Supervision, Validation, Visualization, Writing – Original Draft Preparation, Writing – Review & Editing

## Data availability

### Underlying data

No data are associated with this article.
