# Monitoring of awake bruxism by intelligent app

**DOI:** 10.12688/f1000research.110673.2

**Published:** 2022-12-05

**Authors:** Byron Velásquez Ron, Verónica Mosquera Cisneros, Pamela Pazmiño Troncoso, María Rodríguez Tates, Eddy Alvares Lalvay, Luis Chauca Bajaña, Andrea Ordoñez Balladares

**Affiliations:** 1 Prosthesis Research, Universidad de Las Américas, Quito, Quito, Pichincha, 170523, Ecuador; 2 College of Dentistry, Universidad de Guayaquil, Guayas, 090101, Ecuador

**Keywords:** Awake bruxism; self-report; ecological momentary assessment; smartphone app.

## Abstract

**Background**. Bruxism is a topic of much controversy and is continually debated in the field of dentistry due to the multifaceted clinical relationship that results in painful conditions and consequences to patients. The aim of this review was to determine the effectiveness of a smartphones app in monitoring awake bruxism.

**Methods. **PROSPERO (registration number: CRD42021271190). The eligibility criteria were as followed: observational studies, case–control studies, studies that reported odds ratios, and studies on awake bruxism. The following keywords were searched: [smartphones apps] AND [apps] AND [awake bruxism], OR [sleep bruxism], OR [sleep hygiene], OR [parasomnias], AND [habits].

**Results**. All the included studies found that the use of the smartphone app allows controlled awake bruxism monitoring. The results also show that the slepp bruxism and awake bruxism  are interactive, having negative synergism and substantially increasing the risks of temporomandibular joint pain and temporomandibular disorders.

**Discussion**. In the awake bruxims it was possible to identify 70% symptoms through the different frequencies of behavior provided by the App, within the present technological tools have become daily in young and adult population. The app is effective and easy to use by patients, effectively limiting biases the time of evaluation.

## Introduction

The controversy when talking about bruxism will always be latent among the academy, from a concept of parafunction to a concept of phenomena wherein biological, psychological and exogenous factors act in greater or lesser percentages. ^1^ The independent definitions of day bruxism and night bruxism were pointed out at a meeting of different specialties, with oral rehabilitation experts, maxillofacial surgeons and psychologists, who, in 2020, proposed adequate differentiation between the two. ^2^ Bruxism is a repetitive jaw muscle activity characterized by clenching or grinding of the teeth and/or by bracing or thrusting of the mandible. ^3^^,^^4^ “Bruxism has two distinct circadian manifestations: it can occur during sleep (indicated as sleep bruxism) or during wakefulness (indicated as awake bruxism)”. ^5^^,^^6^ Treatment of bruxism is reduced to several main methods: increasing the vertical dimension of occlusion (VDO) to normal, mesialization of the jaw to influence the position of the articular condyle in the articular fossa and releasing the disc, and positioning the jaw in a balanced stable occlusion. ^7^ This is achieved by splint therapy for a certain period of adaptation and then the result can be fixed by orthodontic treatment, adhesive restorations or prosthetic construction. The stabilizing (occlusal) splint is indicated for the most common symptoms of TMJ and muscle. ^8^^,^^9^ Awake bruxism is currently defined as “masticatory muscle activity during wakefulness that is characterized by repetitive or sustained dental contact and/or reinforcements or pushes of the jaw and is not a movement disorder in healthy individuals”. ^10^

Polysomnography (PSG) and electromyography (EMG) have been used for the evaluation of nocturnal bruxism ^7^; however for the evaluation of awake bruxism (AB), there was no specific evaluator, until 2018 when an app (Manfredini, Bracci, 2018) was created to evaluate and monitor it through the use of smart devices (smartphones). ^11^ Bruxism is not necessarily considered a pathological behavior, but it has clinical consequences, the frequency of AB in the healthy young population allows us to compare with other groups ^12^; in these, psychological factors are determined, including fatigue, muscle pain, tooth wear; having differences between young people and adults differentiating habits and lifestyles that modify the behavior of bruxism. ^13^ The cognitive relationship in oral health suggests a bidirectional causal relationship, there is limited evidence that inflammatory mechanisms, tooth loss, masticatory dysfunction, temporo mandibular joint dysfunction and para functions (bruxism) have the potential to contribute to cognitive decline. ^14^

The use of questionnaires (self-reports), such as clinical observation complemented with electromyography (EMG) have helped in the evaluation of awake bruxism; however, the momentary ecological assessment (EMA) combines real-time approaches to the current state of the patient, which facilitates having an objective assessment. ^15^ The limitations of non-instrumental methods to assess the AB are high and become subjective, the use of EMA allows to collect data in real time for a certain period of time according to the coding of alerts, which are activated according to the daily life of the individual, ^16^ the usefulness in the research field is highlighted when evaluating the oral activity of the individual, unfortunately the data obtained are partial, with little research. ^17^

To limit the bias provided by evaluations of the AB, a group of researchers has introduced an app (BruxApp) for smartphones, its foundation of creation is the implementation of EMA, this collects data through alerts (20 daily) with questions of related conditions simple to accept or deny by the individual: teeth in contact, habits, mandibular hypermobility, clenching and grinding of teeth; characteristic signs of AB. ^18^ It is taken as a starting point young population (young adults) ^19^ whom the researchers determine as the control group, it is monitored by the app for a week (20 daily alerts), the frequency was 28.3% in young people with AB with a low coefficient of variation in jaw muscle activity. ^20^ The objective of the present study was to determine the effectiveness of the smartphone app in monitoring awake bruxism. The PICO question was: is the application of smart apps effective in diagnosing daytime bruxism? P: Smartphone patients with the smart app. I: Intervention of all patients with bruxism C: Comparison of bruxism control with the app versus a control group. O: Observation of the percentage of bruxism control.

## Methods

**Figure 1.**
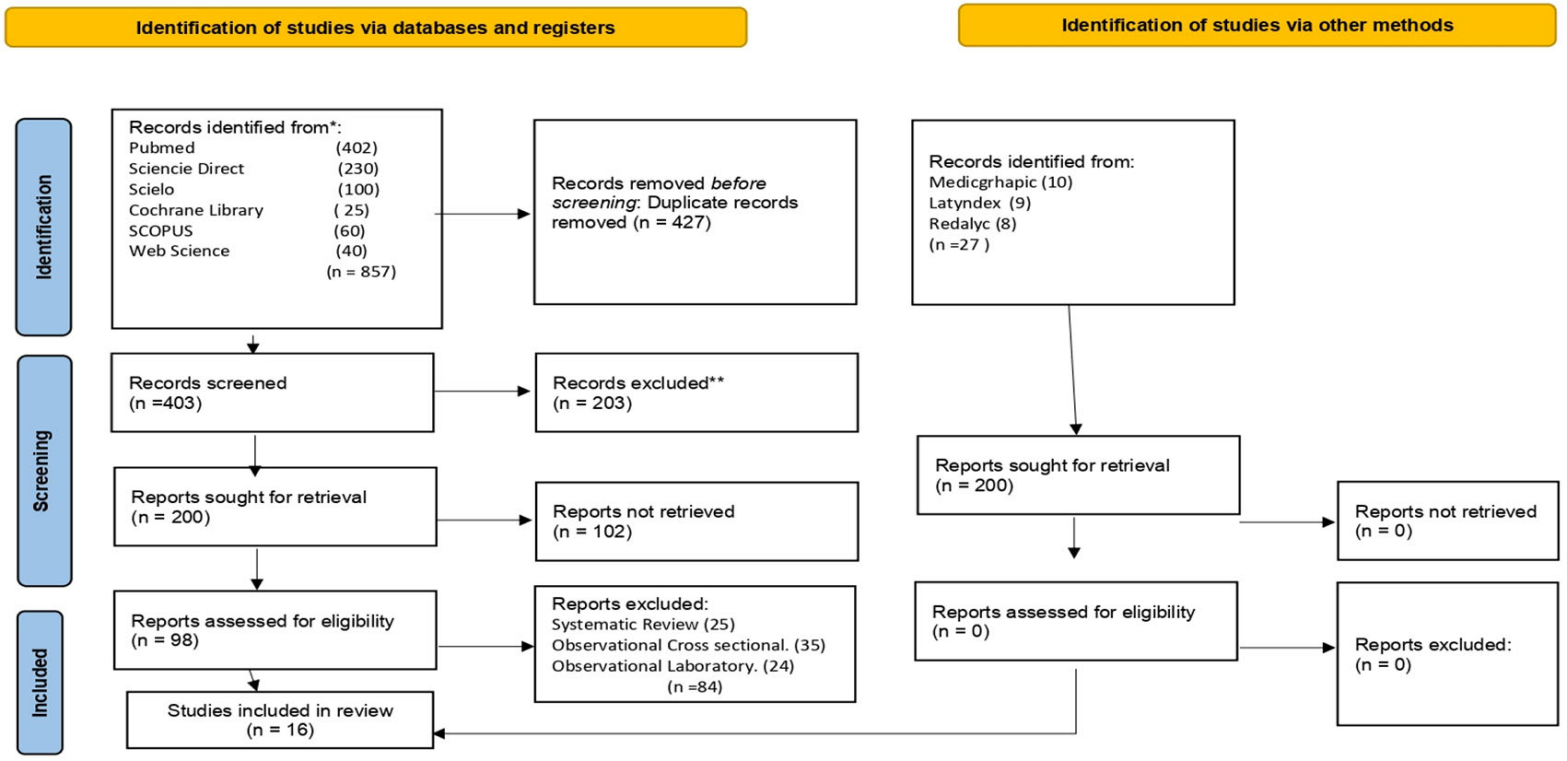
Flow diagram research.

This systematic review was registered with PROSPERO under registration number CRD42021271190. The eligibility criteria were as follows: observational studies, case-control studies, studies that reported odds ratios, and studies on awake bruxism. The following keywords were searched using the Boolean operators AND, OR and NOT: [smartphones apps] AND [apps], [awake bruxism], OR [sleep bruxism], OR [sleep hygiene], OR [parasomnias], OR [habits], OR [chewing], OR [teeth grinding], OR [squeezing teeth], OR [parafunctional habits], OR [parafunctional habit], OR [oral habits] OR [oral habit] OR [oral parafunctional] OR [oral parafunctional] OR [oral parafunctional habit] OR [oral parafunctional] OR [oral parafunctional habit] OR [oral parafunctional] OR [parafunctional oral habit] and [Facial pain] OR [temporomandibular joint disorders] OR [Temporomandibular Joint Dysfunction Syndrome] OR [myofascial pain] OR [syndromes] OR [myalgia]] OR [osteoarthritis] OR [pandemic Cov-19] OR [orofacial pain] OR [orofacial pain] OR [TMD] OR [stress] OR [temporomandibular disorder] OR [myofascial pain] OR [disk displacement] OR [young university] OR [young] OR [adult]. The Scopus, EBSCO, PubMed, Medline Embase, Cochrane Library, and Web of Science databases were searched; alternate databases that were searched included Scielo, Latindex, and Redalyc. Using the PRISMA research protocol, the authors used a flowchart to sequentially explain the selected information. The following complete articles published between January 2014 and June 2021 were included: a total of 857 records were obtained; 27 other records were obtained from other sources; 427 duplicate records were deleted; 200 studies were screened; and 102 records were excluded. In total, 98 studies were included in the qualitative analysis, and 16 studies were includedin the quantitative analysis ( Figure 1).

The authors (BVVR, VMC, PP, LCHB, EDL) independently reviewed the titles and summaries, excluded duplicates and irrelevant articles, and considered only full-text articles. The dates and names of all authors in the final review article were included. Any conflict with respect to the inclusion and exclusion criteria was resolved by the third and fourth authors (MRT, AOB). To control for bias, the Scala JADAD ( Table 1) was used. The data extraction procedure was evaluated according to the criteria of all authors. Articles were classified by the author/year, study objective, study type, methodology, results (standard mean and deviation) and conclusions.

**Table 1.** Jadad Scale for the evaluation of papers.

Study	Study described as randomized	Study appropriate randomization and well described in the article	Study described as double-blind	Double-blind method appropriate	Double-blind description of errors	Total
Flueraşu, M. et al. 2020. ^13^	yes	yes	yes	yes	yes	5
Mir Faeq Ali Quadri, et al. 2015. ^23^	yes	yes	yes	yes	yes	5
Bracci, et al. 2018. ^24^	yes	yes	yes	yes	yes	5
Reissmann, D. et al. 2017. ^25^	yes	yes	yes	yes	no	4
Machado, N. et al. 2020. ^26^	yes	yes	yes	yes	yes	5
Zani, A. et al. 2019. ^31^	yes	yes	yes	yes	yes	5
Shopova, D. et al. 2022. ^32^	yes	yes	yes	yes	yes	5
Sierwald, I. et al. 2015. ^36^	yes	yes	no	yes	yes	4
Wetsellaar, P. et al. 2021. ^44^	yes	yes	yes	yes	yes	5
Wetselaar, P. et al. 2019. ^45^	yes	yes	yes	yes	yes	5
Somay, E. et al. 2020. ^46^	yes	yes	no	yes	yes	4
Zani, A. et al. 2021. ^47^	yes	yes	yes	yes	yes	5
Rofaeel, M. et al. 2021. ^49^	yes	no	yes	yes	yes	4
Serra-Negra, J. et al. 2018. ^50^	yes	yes	yes	yes	yes	5
Winocur, E. et al. 2019. ^51^	yes	no	yes	yes	yes	4
Osiewicz, M. et al. 2019. ^53^	yes	yes	yes	yes	yes	5

## Results

SB is related to nonfunctional occlusion, while AB is related to occlusal interactions, suggesting the need for a different therapeutic approach ( Table 2).

**Table 2.** Summary review.

Article/Author	Country	Objective/Study Type	Participants	Folow up of period	Results	Conclusion
Mir Faeq Ali Quadri, et al. 2015. ^23^	Saudi Arabia	To assess prevalence bruxism among university cross-sectional descriptive.	(95%), 85% 63%, men 22%, women	14 days	Association of stress (p = 0.00; OR = 5.902, 95% CI 2.614–13.325) and khat use (p = 0.05; OR = 1.629, 95% CI 0.360–7.368) with bruxism. Experienced 3.56 times (95% CI; 2.62-11.22) less pain compared.	Bruxism with chewing using Khat (Catha edulis).
Wetselaar, P. et al. 2019. ^45^	Netherlands	To assess the association of TMD pain with awake and sleep bruxism in adults.	733 patients with TMD 890 patients without TMD	2 months	Patients with TMD (33.9%; p<0.001). Patients with TMD (p<0.001). Awake bruxism (OR 1.7; CI 1.0–2.7) sleep bruxism (OR 1.8; CI 1.4–2.4). Awake and sleepy bruxism (OR 7.7; CI 5.4–11.1).	Negative synergism that substantially increases the risk of TMD pain.
Bracci, A. et al. 2018. ^24^	Italy	Awake bruxism behaviors in a sample of healthy young adults using a smartphone-based app for real time Case Control.	46 dental students 15 alerts 1 week	7 days	Relaxed jaw muscles, during Tooth contact (14.5%) jaw clenching (10.0%) the most frequent awake bruxism behaviors.	Contact between teeth grinding of teeth measured as a function od the percentage o f positive alerts during a 1 week.
Reissmann, D. et al. 2017. ^25^	Germany	To explore whether AB and SB interact in AB and SB interact in their associations with painful temporomandibular disorders (TMD).	705 patients	7 days	Awake bruxism (OR = 6.7; 95% CI 3.4 to 12.9) and sleep (OR = 5.1; 95% CI 3.1 to 8.3). multiplicative interaction OR = 057; 95 % CI 0.24 to 1.4 positive additive interaction RERI = 8.6; 95% CI 1.0 to 19.7.	Awake and sleep bruxism are associated with a greater presence of painful.
Flueraşu, M. et al. 2020. ^13^	Rumania	To determine an association between bruxism (sleeping and awake), occlusion (static and dynamic) and pain medications in TMJ in healthy adults Cross-sectional study.	60 subjects 33 women 27 men	1 month	Bruxism than in those without bruxism 3.23 vs 1.46 (p<0.050).	Sleep bruxism is related to non-functional occlusion, while awake bruxism showed occlusal interaction, suggesting the need for a different therapeutic approach.
Machado, N. et al. 2020. ^26^	Brazil	To assess whether the presence of awake bruxism was associated with TMD Cross-sectional study.	56 patients, 58 patients	6 months	The primary effect of awake bruxism was observed when anxiety ANOVA levels of F = 8.61,p = 0.004 and depression ANOVA F = 6.48, p = 0.012 higher and OHRQoL ANOVA F = 8.61, p = 0.04.	Awake bruxism undergoing orthodontic treatment did not develop masticatory muscle.
Wetsellaar, P. et al. 2021. ^44^	Netherlands	To assess the prevalence of awake bruxism and sleep bruxism in the Dutch adolescent population. Case control	920 subjets	12 months	A prevalence of 4.1% and 4.2% was found for awake bruxism and 7.6% and 13.2% for sleep bruxism.	Sleep bruxism is a common condition in the Dutch adolescent population, while awake bruxism is rarer.
Somay, E. et al. 2020. ^46^	Turkey	To assess the prevalence of awake bruxism and sleep bruxism in the Dutch adolescent population. Case control	137 patients 68 hemodial 69 healthy individuals. p < 0.05	6 months	Stress (p = 0.00; OR = 5.902, 95% CI 2.614–13.325) khat use (p = 0.05; OR = 1.629, 95% CI 0.360–7.368) with bruxism khat chewer (95% CI 2.62-11.22)	That hemodialysis patients are more sensitive to TMDs sleep bruxism related dental health problems tha healthy individuals.
Shopova, D. et al. 2022. ^32^	Bulgaria	Complete combined analog and digital clinical protocol in a patient with parafunction.		1 year	The patient was scheduled for periodic monitoring at 3 months. No clinical symptoms of the TMJ were found, the patient also did not report.	Fixed a stable and balanced position of the lower jaw; and repaired the normal physiological position of TMJ.
Rofaeel, M. et al. 2021. ^49^	Canada	Measure massage activity and duration intensity of spontaneous episodes of gritting in healthy individuals with different levels of trait anxiety (TA). Case Control	2993 Israeli high school	1 month	Masseter activity high BP groups (10.23 ± 0.16% MVC) TA groups (8.49 0.16% MVC) low (7.97 ± 0.22% MVC) (all p <0,001).p ≥ 0.05). EMG amplitude of tooth clenching episodes high BP groups (19.97 ± 0.21% CVS) <0, 05).></0, 05).> (16.40 ± 0.24% CVS) low (15.48 ± 0.38% MVC) BP groups (all p</0,001).> The cumulative duration of fist-clenching episodes was not different between groups (p = 0.390).	Among adolescents, sleep and wakefulness bruxism are associated with both emotional aspects and symptoms of facial pain and/or alterations of the masticatory system.
Zani, A. et al. 2021. ^47^	Italy	Assess the frequency of awake bruxism adopting EMA smarthphone-based technology for one week in a sample. Case control	255 people using axiety disorder (AT) score	6 months	The prevalence of bruxism in the two groups (normal and HFS) was not significantly different (p = 0.37). The rate was not significantly different between sleeping and awake bruxism (p = 0.15) in both groups. Stress influenced the occurrence of bruxism in these two groups (p < 0.001).	The intensity of episodes of awake bruxism increases in individuals with a high trait of anxiety.
Serra-Negra, J. et al. 2018. ^50^	Brazil	To assess the association between self-reported awake bruxism (AB) and chronotype profile. Case control	Patients with hemi facial spasms (HFS) for a period of 6 months.	1 week	Awake bruxism 33.7%. chronotype (60.4%), 16.7% had the morning profile. older dental students (OR = 2.640, 95% CI 1.388–5.021) chronotype profile (OR = 3.370, 95% CI 1.302–8.725) with awake bruxism.	The results of this study showed that, although stress has been described as one of the most common aggravating factors in patients with bruxism.
Wetsellaar, P. et al. 2021. ^44^	Netherlands	To assess the prevalence of awake bruxism and sleep bruxism. Case Control	One hundred and fifty-three (N = 153) healthy young adults (mean ± age SD = 22.9 ± 3.2 years).	7 days	A prevalence of 5.0% of the total population was found for awake bruxism and 16.5% for sleep bruxism. As for the five age groups, a prevalence of 6.5%, 7.8%, 4.0%, 3.2% and 3.0%, respectively, was found for awake bruxism, EPISODE computer softwear.	Information on the frequency of different awake bruxism behaviors was provided by adopting the EMA approach. Thanks to the use of Smartphone technology. about 23.6% presented awake bruxism behavior and the most frequent condition was “contact with the teeth”, with a percentage of 13.6%.
Winocur, E. et al. 2019. ^51^	Israel	To determine the emotional, behavioral, and physiological associations of sleep and awakened bruxism among Israeli adolescents. Case Control	255 patients	1 year	(43.4%) bruxism (34.5%) awake bruxism, (14.8%) sleep bruxism, and (7.3%) both sleep and awake bruxism. Odds Ratios (OR) of 1.38, 2.08 and 2.35, respectively). Stress increased the risk of SB by 3.2%, temporomandibular symptoms (OR = 2.17) and chewing difficulties (OR = 2.35). Neck pain showed a negative association (OR = 0.086). anxiety (OR = 1.6).	Anxiety is considered an important trait in patients suffering from awake bruxism. Electromyography is used to measure episodes of spontaneous tooth tightening during wakefulness, it was shown that healthy individuals with a high and clinically relevant anxiety trait have increased mass activity and more intense spontaneous episodes of teeth clenching upon awakening.
Zani, A. et al. 2019. ^31^	Italy	Evaluation EMI vs Smarthphone app in Italian young population. Cross Sectional	205 dental students	7 days	T1:62% relaxed jaw muscles 20 % contact with teeth 14% braces awake behaviors T2:74% relaxed jaw muscles 11% contact with teeth 13% braces awake behaviors	Students over the age os 22 and those with the evening chronotype profile were the most likely to suffer from sleep bruxism.
Osiewicz, M. et al. 2019. ^53^	Italy	Describe the process of understanding the BruxApp smartphone application in the context of an ongoing multicenter project on the epidemiology of awakened bruxism (AB). Case Control	Sample of healthy young adults, dental students from 11 universities.	7 days	There are two software versions available, namely BruxApp and BruxApp Research. For both versions, a reverse translation was performed from Polish to English to verify the accuracy of the translation procedure.	Students over the age os 22 and those with the evening chronotype profile were the most likely to suffer from sleep bruxism.

## Discussion

All the authors agree that the use of the smartphone app allows controlled AB monitoring by the patient. The current study also showed that the two bruxism are interactive, with negative synergism substantially increasing the risks of TMJ pain and TMD. Signs such as contact between the teeth, clenching of teeth, teeth grinding, and jaw clenching are well defined in the application in AB, it was possible to identify 70% symptoms through the different frequencies of behavior provided by the app, within the present technological tools have become daily in young and adult population. ^21^ In the studies reviewed, the EMA was clear for the entire assigned sample. ^22^^,^^23^ In the studies that entered the analysis, the six conditions indicated by the application menu were investigated, relaxed jaw muscles (non-contact teeth), teeth in contact (sander in fixed position), mandibular clenching (without contact between the teeth), dental clenching (strong contact in fixed position), dental grinding and area of pain (temporary, interciliary, temple, preauricular, auricular, mandibular angle, mentonian, neck, frontal, infra and supraorbital), ^24^^,^^25^ the data that were obtained were handled by the application menu that allowed to precisely extract a Microsoft Excel file (20 alerts × 7 days) in real time. ^26^ The limitation that was found in the present systematic review is the difficulty of comparing with other studies by the different experimental designs (retrospective), while to apply self-reports are unique times. ^27^ By assessing population behavior frequency is the baseline for observational EMA studies that aids massive data collection, ^28^ it also helps to compare findings related to dietary habits, smoking, medications, psychological pathologies, and comorbid conditions. ^29^ Some studies take as a control group young population Kardeş and Kardeş 2019 analyzed healthy young population finding dental contact (13.6%), teeth grinding (0.5%) and relaxed jaw muscles (76.4%), with a combined frequency of AB of (23.6%). ^30^ Some studies take as a control group young population Bracci *et al*. 2018 analyzed healthy young population finding dental contact (13.6%), teeth grinding (0.5%) and relaxed jaw muscles (76.4%), with a combined frequency of AB of 23.6%. ^31^ These results could be considered a reference point for future research on the epidemiological characteristics of AB in healthy young adults, young people with pathologies, adults and geriatric patients. ^32^^,^^33^ The importance of psychological factors was determined, well-defined changes after the COVID-19 pandemic, having been analyzed in AB, the findings were that females are more likely to experience stress, compared with males, the explanation women report better about their emotions ^34^ but the depressive state leads to generate AB crisis with BS in the two genders due to the socio-economic conditions generated by the pandemic, it should be clarified that previous systematic reviews found no gender differences in the frequency of AB ^35^ which contrasts with current information. ^36^ No significant differences were found in the university population, young adults, some authors point out that the monitoring could have been carried out in transition for the student population so that high stress was not indicated, it would be important to develop future research in times such as semester evaluations to determine significant differences. ^37^ It should be considered that the elaboration of the self-report must be controlled, so that unnecessary biases are avoided, for this reason the calibration of the instrument is essential whether individual or group, avoiding or reducing homogeneity to a minimum, ^38^^,^^39^ through training and socialization that allows the population to understand the reliable use of self-report based on EMA. ^40^ The characteristics of the populations studied directly influence the results, the age factor, educational level, work activity, socioeconomic status are aspects that influence in substance. ^41^^,^^42^ Camara M, *et al*. 2020 found that in one week the relaxation of the mandibular muscles was very low, they conclude that not only in healthy young population the symptoms change from one day to the next, ^43^ the population comportment must be specific, this makes variable the behavior of the AB monitored with the app, recognizing natural fluctuation and difficulty in recognizing the symptoms. ^44^ Muscle relaxation can be recognized by the individual, also clenching of teeth, ^45^ can be a good reference to evaluate the behavior of AB to be a conscious and controlled activity, ^46^ other authors indicate that the use of SMEs provides reliability in the monitoring of AB, the reason lowers the influence of natural fluctuation that the population presents regardless of age or gender. It is recommended to conduct future research that considers long-term monitoring of AB, the hypothesis should be tested that the manifestations of AB: relaxed jaw muscles (non-contact teeth), teeth in contact (sander in fixed position), mandibular clenching (no contact between teeth), dental clenching (strong contact in fixed position), dental grinding and area of pain (temporary, interciliary, temple, preauricular, auricular, mandibular angle, mentonian, neck, frontal, infra and supraorbital, clinical consequences such as temporo mandibular joint dysfunction, regional myalgias ^47^ are determined. Continuing with the technological line, the effectiveness of an email-based registration and recovery system should be studied if the individual detects non-functional diurnal contact or muscle contracture, an effective strategy for the treatment of temporo mandibular disorders. ^48^ An assessment of the associated factors and conditions can, in theory, increase or decrease the frequencies of AB behaviors in the app monitored population based on the EMA self-report ( *e.g.*, dietary, or smoking habits, medication use, psychological problems, and comorbid conditions). ^50^^,^^51^ Data can be added to ongoing studies that consider the 2018 definition of bruxism ^52^ and the refinement of assessment strategies. Comparisons between populations are necessary and can be used in the context of an ongoing multicenter project on the epidemiology of bruxism. ^53^

## Conclusions

The app used to monitor awake bruxism is effective, and its ease of use allows a fundamental approach to diagnosis. It should be noted that the use of the App allows us to monitor the variable behavior of awake bruxism.

## Author contributions

**Velasquez B:** Conceptualization, Data Curation, Formal Analysis, Investigation, Methodology, Project Administration, Resources, Validation, Visualization, Writing Original Draft Preparation. Writing -review & Edith.

**Alvarez E.:** Conceptualization, Data Curation, Formal Analysis, Investigation, Methodology, Project Administration, Resources

**Mosquera V:** conceptualization, Data Curation, Formal Analysis, Investigation, Methodology, Project Administration, Resources

**Pazmiño P:** conceptualization, Data Curation, Formal Analysis, Investigation, Methodology, Project Administration, Resources

**Rodriguez M:** Conceptualization, Data Curation, Validation, Visualization, Writing – Original Draft Preparation, Writing – Review & Editing

**Chauca L:** Formal Analysis, Resources, Supervision, Validation, Visualization, Writing – Original Draft Preparation, Writing – Review & Editing

**Ordoñez A:** Formal Analysis, Resources, Supervision, Validation, Visualization, Writing – Original Draft Preparation, Writing – Review & Editing

## Data availability

### Underlying data

No data are associated with this article.
