# Supplementary material for: Monitoring of awake bruxism by intelligent app
Source: F1000Res. 2022 Dec 5;11:479. Originally published 2022 Apr 29. [Version 2] doi: 10.12688/f1000research.110673.2 (PMC9768384; doi:10.12688/f1000research.110673.2)
Supplement: Peer review history

# Peer review: Reviewer response for version 2

**DOI:** 10.5256/f1000research.140127.r157372

**Published:** 2022-12-20

**Authors:** Dobromira Shopova

**Affiliations:** 1 Medical University of Plovdiv, Plovdiv, Bulgaria

The article is of suitable format, design and content to be published in its current form after the corrections made.

Are the rationale for, and objectives of, the Systematic Review clearly stated?

Partly

Is the statistical analysis and its interpretation appropriate?

I cannot comment. A qualified statistician is required.

Are sufficient details of the methods and analysis provided to allow replication by others?

Partly

Are the conclusions drawn adequately supported by the results presented in the review?

No

Reviewer Expertise:

I am a specialist in prosthetic dentistry and in recent years I have been dealing with modern methods for the treatment of bruxism.

I confirm that I have read this submission and believe that I have an appropriate level of expertise to confirm that it is of an acceptable scientific standard.

# Peer review: Reviewer response for version 2

**DOI:** 10.5256/f1000research.140127.r157373

**Published:** 2022-12-19

**Authors:** Nithin Manchery

**Affiliations:** 1 School of Dentistry, The University of Queensland, Brisbane, Qld, Australia

The authors have addressed the comments.

Are the rationale for, and objectives of, the Systematic Review clearly stated?

Yes

Is the statistical analysis and its interpretation appropriate?

Yes

Are sufficient details of the methods and analysis provided to allow replication by others?

Yes

Are the conclusions drawn adequately supported by the results presented in the review?

Partly

Reviewer Expertise:

General dentistry and older adults.

I confirm that I have read this submission and believe that I have an appropriate level of expertise to confirm that it is of an acceptable scientific standard.

# Peer review: Reviewer response for version 1

**DOI:** 10.5256/f1000research.122302.r150308

**Published:** 2022-10-19

**Authors:** Nithin Manchery

**Affiliations:** 1 School of Dentistry, The University of Queensland, Brisbane, Qld, Australia

Overall this is an interesting topic of research. A few suggestions for the authors to consider are provided below.

 Abstract.

- Results section - Suggest rephrasing, " All the authors agree..." to " All the included studies found..."
- The sentence " The results also show that...the two bruxism..." is unclear. Please check the sentence. 
- Kindly define/write the words in full and provide abbreviations within brackets before using them elsewhere in the text (e.g., in the discussion section of the abstract: "AB").

Introduction

- Suggest the knowledge gap could be elaborated a little to strongly justify the rationale for this review (e.g. the data are only partial and little research - what were the findings and how did they compare, are there only the 2 studies so far?).
- Also, to include the potential implications of the findings from this SR.

Methods

- Were articles restricted only to English? Was this limit applied?
- In the sentence, "...98 studies were included in the qualitative analysis...", suggest changing to '98 studies were assessed for eligibility...' for better clarity.

Results

- Consider explaining the results in detail in word. The basic characteristics, risk of bias, key findings.
- Was a meta-analysis considered or avoided due to heterogenicity between the included studies?

Discussion

- The authors constantly compare findings between the 2 types of bruxisms. I suppose this deviates from the main aim - which is only focussed on AB. 
- Are all the limitations addressed? Bias, strength of evidence.

Conclusion

- Please add the key findings from the SR and significance of the findings.

Are the rationale for, and objectives of, the Systematic Review clearly stated?

Yes

Is the statistical analysis and its interpretation appropriate?

Yes

Are sufficient details of the methods and analysis provided to allow replication by others?

Yes

Are the conclusions drawn adequately supported by the results presented in the review?

Partly

Reviewer Expertise:

General dentistry and older adults.

I confirm that I have read this submission and believe that I have an appropriate level of expertise to confirm that it is of an acceptable scientific standard, however I have significant reservations, as outlined above.

# Response

**Published:** 2022-10-19

**Authors:** Byron Velasquez Ron

**Affiliations:** Universidad de Las Américas, Quito, Ecuador

Thanks so much for your commentaries, in this days working with my team, correct the papers and send with corrections.

 Regards. 

 Byron

# Peer review: Reviewer response for version 1

**DOI:** 10.5256/f1000research.122302.r145993

**Published:** 2022-09-30

**Authors:** Dobromira Shopova

**Affiliations:** 1 Medical University of Plovdiv, Plovdiv, Bulgaria

The introduction is short, with only 17 cited authors. In general, the number of references is not large - 50, which tends to the minimum for a review article. I ask the authors to add more articles, especially those published in the last 5 years. If desired, they can use some of those that I consider appropriate:

1. Shopova D, Bozhkova T, Yordanova S and Yordanova M. Case Report: Digital analysis of occlusion with T-Scan Novus in occlusal splint treatment for a patient with bruxism [version 2; peer review: 2 approved]. *F1000Research* 2022, **10**:915 ^1^ – It presents a digital device of measurement of occlusal forces, which is very useful in nowadays practice.
2. Shopova D and Mladenov K. Case Report: A digital workflow in the treatment of bruxism in a young patient [version 2; peer review: 2 approved]. *F1000Research* 2022, **10**:894 ^2^ – It presents the complete procedure of diagnosis and treatment in case of bruxism only by digital workflow.
3. Bozhkova, T., & Shopova, D. (2021). T-Scan Novus System in the Management of Splints—Pilot Study. *European Journal of Dentistry* ^3^. – Again, digital method of measurement of occlusal forces, but with clinical examples.
4. Shopova, D., Yordanova, M., & Yordanova, S. (2021). Software Details in Occlusal Splint Creation through 3Shape Design Studio. *Open Access Macedonian Journal of Medical Sciences*, *9*(D), 330-335 ^4^. – The treatment plan is very important. The dentist should know the opportunities of digital software, to know how they can help to the patient in every different situation.
5. Shopova, D., Bakova, D., Yordanova, M., & Yordanova, S. (2021). TELEDENTISTRY METHODS IN ORTHODONTICS AND PROSTHETIC DENTISTRY DURING COVID-19 PANDEMIC. *KNOWLEDGE-International Journal*, *49*(4), 667-672. – This article presents digital approach of bruxism treatment again, but this time from pandemic point of view.
6. Shopova, D., Yordanova, M., & Yordanova, S. (2021). 3Shape Digital Design Software in Splints Creation—A Pilot Study. *European Journal of Dentistry* ^5^ *. *– here it presents the difference in occlusal surfaces in splints, which every dentist has to know, not to leave the choice in dental technicians’ hands.
7. Taneva, I., Grozdanova-Uzunova, R., & Uzunov, T. (2021, March). Occlusal splints–changes in the muscular activity. In *Journal of Physics: Conference Series* (Vol. 1859, No. 1, p. 012046). IOP Publishing ^6^. – The condition of the muscles is very important for the successful result of the treatment in bruxism.
8. Taneva, I., Uzunov, T., & Milanov, N. (2020). Complete digital approach for bruxism management. *Problems of Dental Medicine*, *46*, 18-27. – It presents modern method of bruxism treatment, very similar with the purpose of the reviewed article.

All of these articles presented the modern methods of diagnosis and treatment in bruxism cases. They can be find in Google scholar or linked to this report, some of them are uploaded in Scopus or Web of Science. Authors can add and more similar articles to enrich their article. 50 cited articles are too small number.

 Long tables make the results quite difficult. Try to systematize the cited authors more tightly.

 The discussion is voluminous enough and a sufficient number of authors are compared.

 The conclusion consists of one short sentence. Authors can submit their opinion on the most suitable app according to them.

Are the rationale for, and objectives of, the Systematic Review clearly stated?

Partly

Is the statistical analysis and its interpretation appropriate?

I cannot comment. A qualified statistician is required.

Are sufficient details of the methods and analysis provided to allow replication by others?

Partly

Are the conclusions drawn adequately supported by the results presented in the review?

No

Reviewer Expertise:

I am a specialist in prosthetic dentistry and in recent years I have been dealing with modern methods for the treatment of bruxism.

I confirm that I have read this submission and believe that I have an appropriate level of expertise to confirm that it is of an acceptable scientific standard, however I have significant reservations, as outlined above.

## References

[1] : Case Report: Digital analysis of occlusion with T-Scan Novus in occlusal splint treatment for a patient with bruxism. *F1000Research* .2022;10: 10.12688/f1000research.72951.2 10.12688/f1000research.72951.2 PMC875897235083036

[2] : Case Report: A digital workflow in the treatment of bruxism in a young patient. *F1000Research* .2022;10: 10.12688/f1000research.72961.2 10.12688/f1000research.72961.2 PMC944556536111070

[3] : T-Scan Novus System in the Management of Splints-Pilot Study. *Eur J Dent* .2022;16(2) : 10.1055/s-0041-1736315 454-457 10.1055/s-0041-1736315 34863081PMC9339920

[4] : Software Details in Occlusal Splint Creation through 3Shape Design Studio. *Open Access Macedonian Journal of Medical Sciences* .2021;9(D) : 10.3889/oamjms.2021.7870 330-335 10.3889/oamjms.2021.7870

[5] : 3Shape Digital Design Software in Splints Creation-A Pilot Study. *Eur J Dent* .2021; 10.1055/s-0041-1739546 10.1055/s-0041-1739546 34952555PMC9683882

[6] : Occlusal splints – changes in the muscular activity. *Journal of Physics: Conference Series* .2021;1859(1) : 10.1088/1742-6596/1859/1/012046 10.1088/1742-6596/1859/1/012046

# Response

**Published:** 2022-10-03

**Authors:** Byron Velasquez Ron

**Affiliations:** Universidad de Las Américas, Quito, Ecuador

Good morning dear reviewer, we will gladly make the changes as soon as possible from your valuable comments.

 Kind regards.

 Byron
